# The unfolded protein response components IRE1α and XBP1 promote human coronavirus infection

**DOI:** 10.1128/mbio.00540-23

**Published:** 2023-06-12

**Authors:** Jessica M. Oda, Andreas B. den Hartigh, Shoen M. Jackson, Ana R. Tronco, Susan L. Fink

**Affiliations:** 1 Department of Laboratory Medicine and Pathology, University of Washington, Seattle, Washington, USA; University of Pennsylvania, Philadelphia, Pennsylvania, USA; University of Maryland School of Medicine, Baltimore, Maryland, USA

**Keywords:** coronavirus, unfolded protein response, ER stress, SARS-CoV-2, HCoV-OC43, endoplasmic reticulum, IRE1α, XBP1

## Abstract

The cellular processes that support human coronavirus replication and contribute to the pathogenesis of severe disease remain incompletely understood. Many viruses, including coronaviruses, cause endoplasmic reticulum (ER) stress during infection. IRE1α is a component of the cellular response to ER stress that initiates non-conventional splicing of *XBP1* mRNA. Spliced *XBP1* encodes a transcription factor that induces the expression of ER-related targets. Activation of the IRE1α–XBP1 pathway occurs in association with risk factors for severe human coronavirus infection. In this study, we found that the human coronaviruses HCoV-OC43 (human coronavirus OC43) and SARS-CoV-2 (severe acute respiratory syndrome coronavirus-2) both robustly activate the IRE1α–XBP1 branch of the unfolded protein response in cultured cells. Using IRE1α nuclease inhibitors and genetic knockdown of IRE1α and XBP1, we found that these host factors are required for optimal replication of both viruses. Our data suggest that IRE1α supports infection downstream of initial viral attachment and entry. In addition, we found that ER stress–inducing conditions are sufficient to enhance human coronavirus replication. Furthermore, we found markedly increased XBP1 in circulation in human patients with severe coronavirus disease 2019 (COVID-19). Together, these results demonstrate the importance of IRE1α and XBP1 for human coronavirus infection.

**IMPORTANCE:**

There is a critical need to understand the cellular processes co-opted during human coronavirus replication, with an emphasis on identifying mechanisms underlying severe disease and potential therapeutic targets. Here, we demonstrate that the host proteins IRE1α and XBP1 are required for robust infection by the human coronaviruses, SARS-CoV-2 and HCoV-OC43. IRE1α and XBP1 participate in the cellular response to ER stress and are activated during conditions that predispose to severe COVID-19. We found enhanced viral replication with exogenous IRE1α activation, and evidence that this pathway is activated in humans during severe COVID-19. Together, these results demonstrate the importance of IRE1α and XBP1 for human coronavirus infection.

## INTRODUCTION

Coronaviruses are a family of enveloped, positive-stranded RNA viruses, including the recently emergent, currently pandemic, severe acute respiratory syndrome coronavirus-2 (SARS-CoV-2). Coronavirus replication utilizes incompletely understood host cell pathways and relies on intracellular membranes derived from the endoplasmic reticulum (ER) (1). Disruption of the normal ER environment causes a state termed ER stress, which is detected by the cellular unfolded protein response (2). Inositol-requiring enzyme 1α (IRE1α) is a component of the unfolded protein response and ER-resident transmembrane protein that oligomerizes and autophosphorylates during ER stress (3, 4). This activates its cytosolic RNase domain to initiate non-conventional splicing of the mRNA encoding X-box binding protein-1 (XBP1). Spliced *XBP1* mRNA is a specific product of activated IRE1α and, when translated, encodes a transcription factor that upregulates genes, including those involved in ER function (5, 6). IRE1α also targets other specific RNAs, leading to their degradation in a process termed regulated IRE1-dependent decay (RIDD) (7). IRE1α activation occurs in association with risk factors for severe SARS-CoV-2 infection, including diabetes, hypertension, aging, and obesity (8 - 10).

Some coronaviruses manipulate IRE1α for their own benefit. Transmissible gastroenteritis virus (TGEV) is a porcine alphacoronavirus that activates IRE1α, leading to the induction of XBP1 targets (11). In TGEV-infected cells, IRE1α also cleaves a microRNA that regulates type I interferon receptor signaling, leading to the evasion of interferon responses. IRE1α activation and XBP1 target gene induction also occur during infection with the avian gammacoronavirus and infectious bronchitis virus (IBV) (12). Similar to our findings with hepatitis C virus (13), IRE1α plays an anti-apoptotic role during IBV infection (12). Murine hepatitis virus is a betacoronavirus that activates IRE1α (14, 15). However, XBP1 target genes are not induced (14) or only slightly elevated (16). Unlike these non-human pathogens, SARS coronavirus, a human betacoronavirus closely related to SARS-CoV-2, limits IRE1α activation and *XBP1* splicing in infected cells (17, 18).

In this study, we examined the role of IRE1α and XBP1 during infection with the human betacoronaviruses, HCoV-OC43 and SARS-CoV-2. We found that IRE1α is activated and promotes infection via XBP1 during infection of cultured cells with both viruses. We demonstrated that pre-existing ER stress and activation of IRE1α resulted in heightened human coronavirus infection, and elevated XBP1(S) protein is present in samples from patients with severe COVID-19. Together, these findings reveal that IRE1α and XBP1 are cellular host factors that promote the replication of the human coronaviruses HCoV-OC43 and SARS-CoV-2.

## RESULTS

### Human coronaviruses activate IRE1α and induce XBP1 targets

**Fig 1:**
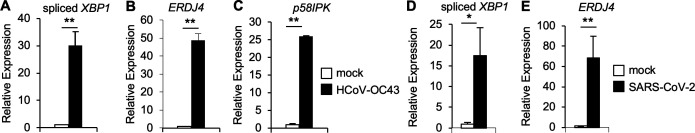
Human coronavirus infection activates IRE1α and induces XBP1 targets. (**A–C**) HCT-8 cells were infected with HCoV-OC43 for 48 hours. (D+**E**) Calu-3 cells were infected with SARS-CoV-2 for 48 hours. The relative abundance of spliced *XBP1* (A+**D**), *ERDJ4* (B+**E**), and *p58IPK* (**C**) were determined by quantitative RT-PCR. Data are means ± SD of four replicates and are representative of three independent experiments. *P < 0.05, **P < 0.01 by unpaired *t*-test.

The human betacoronavirus SARS-CoV inhibits IRE1α activation resulting in minimal *XBP1* splicing during infection (17, 18). To determine whether other human betacoronaviruses similarly limit the activation of the IRE1α branch of the unfolded protein response, we infected human HCT-8 epithelial cells with HCoV-OC43 and assessed *XBP1* mRNA splicing using qRT-PCR with the primers specific for spliced *XBP1* mRNA. We found that HCoV-OC43 infection stimulated robust *XBP1* splicing in this system (Fig. 1A), consistent with findings in infected neurons (19). The model betacoronavirus murine hepatitis virus activates IRE1α, but XBP1 target genes are not induced (14) or only slightly elevated (16). Induction of *ERDJ4* and *P58IPK* requires XBP1 (20, 21), and we found strong expression of these XBP1-responsive genes in HCoV-OC43-infected cells (Fig. 1B and C).

In some settings, IRE1α’s RNase domain also degrades host RNAs including *BLOC1S1*, *SCARA3*, *COL6A1*, and *HGSNAT* through the process known as RIDD (22). Consistent with observations in other cell types (23), these RIDD targets were reduced in HCT-8 cells treated with the ER stress–inducing agent, tunicamycin (Fig. S1A). However, *BLOC1S1*, *SCARA3*, *COL6A1*, and *HGSNAT* were unaffected by HCoV-OC43 (Fig. S1B), suggesting that IRE1α activity is limited to *XBP1* splicing during infection.

To determine whether SARS-CoV-2 similarly activates IRE1α and induces XBP1-dependent gene expression, we infected human Calu-3 lung epithelial cells with the clinically derived USA-WA1/2020 SARS-CoV-2 strain. SARS-CoV-2 infection stimulated *XBP1* splicing (Fig. 1D) and expression of the XBP1-responsive gene, *ERDJ4* (Fig. 1E). However, the RIDD targets *BLOC1S1*, *SCARA3*, *COL6A1*, and *HGSNAT* were not reduced (Fig. S1C), similar to our findings with HCoV-OC43 infection. Together these results indicate that the human betacoronaviruses SARS-CoV-2 and HCoV-OC43 both activate IRE1α to splice *XBP1* mRNA, leading to the expression of XBP1-responsive genes, but RIDD targets are not degraded.

### IRE1α is required for optimal HCoV-OC43 infection

**Fig 2:**
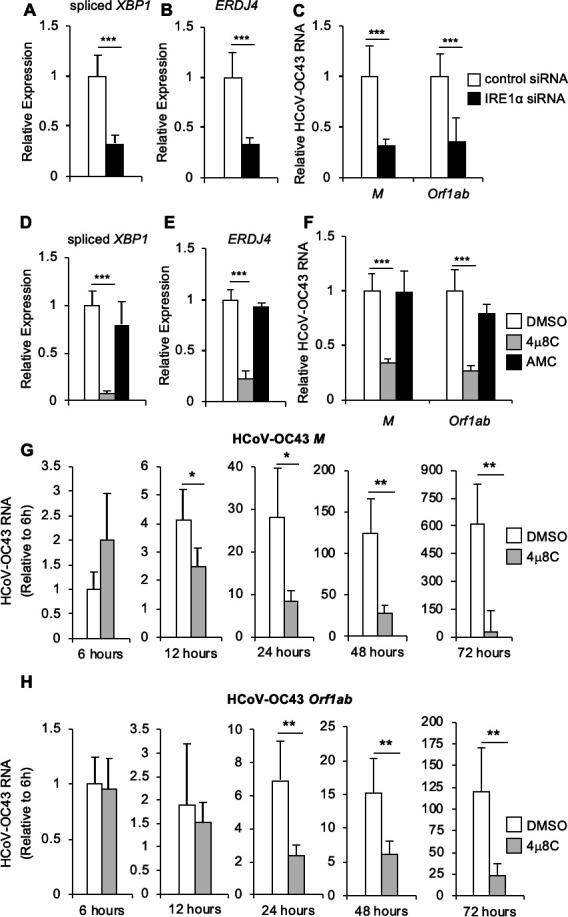
IRE1α is required for optimal HCoV-OC43 infection. (**A–C**) HCT-8 cells were transfected with siRNA targeting IRE1α or non-targeting control siRNA and then infected with HCoV-OC43 at an MOI of 0.01. RNA was harvested 48-hour post-infection, and the relative abundance of spliced *XBP1* (**A**), *ERDJ4* (**B**), and HCoV-OC43 viral RNA (**C**) were determined by quantitative RT-PCR. (**D–H**) HCT-8 cells were treated with IRE1α nuclease inhibitor 4μ8C, structurally similar negative control AMC or DMSO solvent control prior to infection with HCoV-OC43 at an MOI of 0.01 (**D–F**) or an MOI of 1 (**G–H**). RNA was harvested 48-hour post-infection (**D–F**) or at the indicated time point (**G and H**), and the relative abundance of spliced *XBP1* (**D**), *ERDJ4* (**E**), and HCoV-OC43 viral RNA (**F–H**) was determined by quantitative RT-PCR. HCoV-OC43 viral RNA abundance at the indicated time points (**G and H**) is calculated relative to the first time point. Data are means ± SD of six (**A–C**) or four (**D–H**) replicates and are representative of three (**A–F**) and two (**G and H**) independent experiments, respectively. *P < 0.05, **P < 0.01, ***P < 0.001 by unpaired *t*-test.

IRE1α promotes infection by a number of RNA viruses, including hepatitis C (13), Zika (20), and influenza A (24). To determine the role of this host factor during HCoV-OC43 infection, we used small-interfering RNA (siRNA) to knock down IRE1α in HCT-8 cells. As a functional control for IRE1α inactivation, we assessed *XBP1* mRNA splicing (Fig. 2A) and sXBP1 protein (Fig. S2A and B) and found both were reduced in cells treated with siRNA targeting IRE1α. Consistently, XBP1-dependent *ERDJ4* expression in HCoV-OC43-infected cells was also reduced by siRNA targeting IRE1α (Fig. 2B). We then assessed the effect of IRE1α knockdown on viral replication and found reduced HCoV-OC43 RNA, using qRT-PCR with two different primer sets to quantify viral RNA (Fig. 2C). These results suggest that HCoV-OC43 requires IRE1α for efficient viral infection.

IRE1α affects multiple cellular pathways via its kinase and nuclease functions (2). To determine whether IRE1α’s RNase activity promotes HCoV-OC43 infection, we treated cells with the selective IRE1α nuclease inhibitor, 4μ8C (25) and structurally similar inactive control molecule AMC (13, 20). We found that 4μ8C, but not the inactive control AMC, prevented *XBP1* mRNA splicing (Fig. 2D), sXBP1 protein expression (Fig. S2C), and induction of the XBP1 target *ERDJ4* (Fig. 2E) verifying its effect on IRE1α. Inhibiting IRE1α’s nuclease activity with 4μ8C significantly reduced the abundance of HCoV-OC43 viral RNA (Fig. 2F), indicating that the RNase activity is required for optimal infection. We then tested a structurally distinct IRE1α nuclease inhibitor, STF-083010 (26), which also inhibited *XBP1* mRNA splicing (Fig. S3A), sXBP1 protein expression (Fig. S2C), *ERDJ4* induction (Fig. S3B), and reduced HCoV-OC43 viral RNA (Fig. S3C).

These experiments used a low multiplicity of infection (MOI) of 0.01, so to further characterize the requirement for IRE1α throughout a high MOI infection, we performed a time course experiment with an MOI of 1. We found that at early time points during HCoV-OC43 infection, there was no significant decrease in viral RNA in the presence of IRE1α inhibitor compared to dimethylsulfoxide (DMSO) control (Fig. 2G and H). However, at 24-hour post-infection and later time points, viral RNA was significantly reduced with IRE1α inhibition (Fig. 2G and H), similar to our findings from the low MOI infection model.

**Fig 3:**
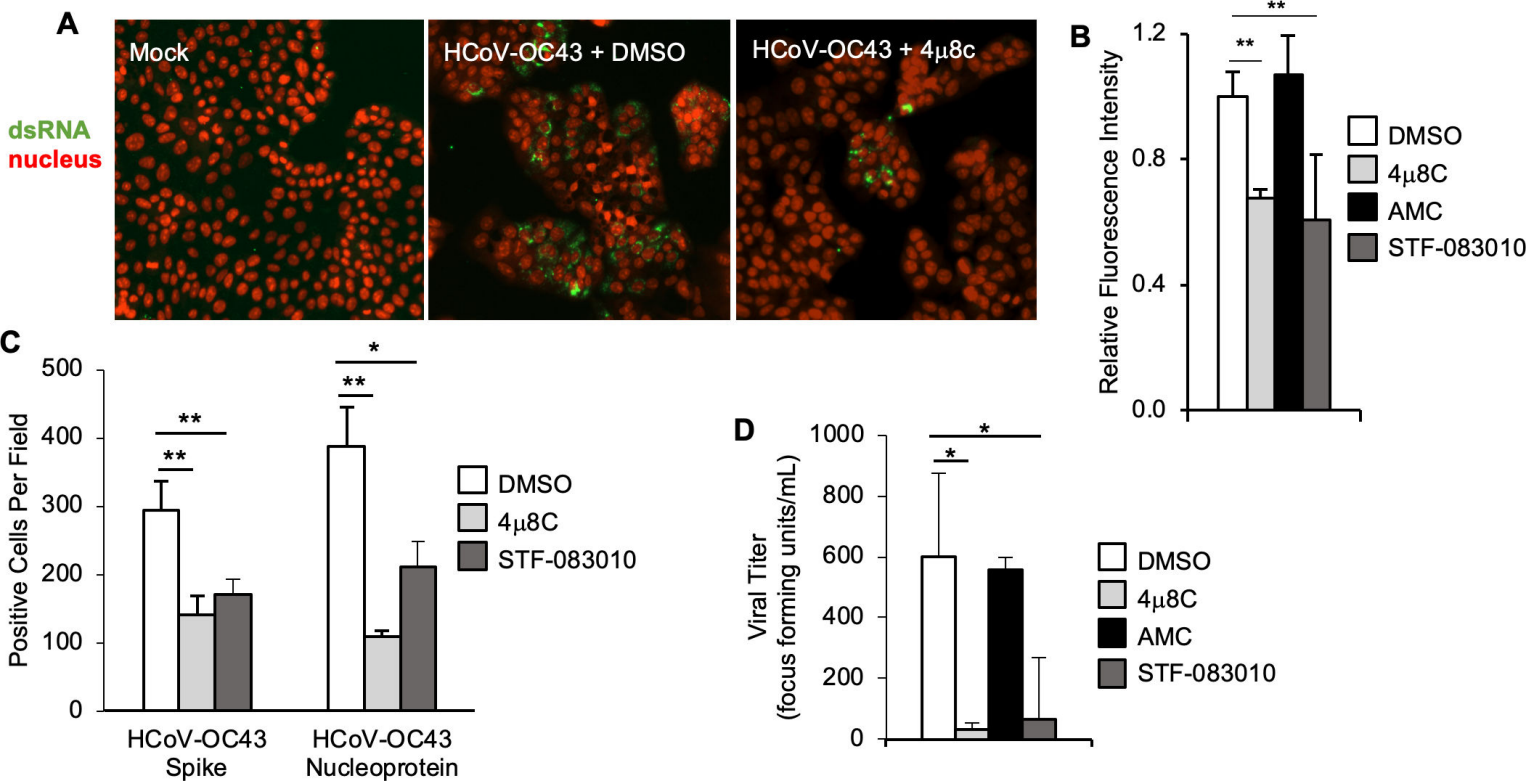
IRE1α is required for optimal HCoV-OC43 infection assessed by immunofluorescence and titer. (**A–D**) HCT-8 cells were treated with IRE1α nuclease inhibitor 4μ8C, structurally similar negative control AMC, IRE1α nuclease inhibitor STF-083010 or DMSO solvent control prior to infection with HCoV-OC43. (**A–C**) Cells were fixed 48-hour post-infection. (**A**) Double-stranded RNA (dsRNA, green) was visualized by immunostaining, and nuclei were counterstained with TO-PRO-3 (red). (**B**) Relative total fluorescence intensity was calculated for dsRNA. (**C**) HCoV-OC43 viral proteins spike and nucleoprotein were visualized by immunostaining, and positive cells were quantified per field at 10× magnification. (**D**) Viral supernatant was harvested 48-hour post-infection, then diluted serially and plated on HCT-8 cells for an endpoint focus-forming assay. Data are means ± SD of six (C) or four (C+D) replicates and are representative of three (A+B) and two (C+D) independent experiments, respectively. *P < 0.05, **P < 0.01 by unpaired *t*-test.

To assess viral RNA using an orthogonal approach, we performed immunostaining for the viral replication intermediate, double-stranded RNA (dsRNA) (Fig. 3A). We found that the selective IRE1α nuclease inhibitors 4μ8C and STF-083010 both reduced dsRNA staining (Fig. 3A and B). We next used immunostaining to assess viral spike and nucleoprotein production (Fig. S4A). IRE1α nuclease inhibition with 4μ8C and STF-083010 also significantly reduced viral protein, compared to DMSO solvent control (Fig. 3C; Fig. S4A). Finally, we collected supernatant from infected cells and determined infectious viral titer using an endpoint focus-forming assay. We found that both IRE1α inhibitors, but not the inactive AMC control, resulted in a significant reduction in infectious HCoV-OC43 viral titer (Fig. 3D). Together, these findings demonstrate that HCoV-OC43 infection requires IRE1α RNase activity for optimal replication.

We previously found that IRE1α promotes the replication of hepatitis C virus by blocking apoptosis in infected cells (13). Inhibition of IRE1α sensitized HCV-infected cells to death, limiting viral replication at the later stages of infection. IRE1α also plays an anti-apoptotic role during infection with IBV (12). Based on these findings, we hypothesized that inhibiting IRE1α may sensitize HCoV-OC43-infected cells to die, thus terminating viral replication. To test this hypothesis, we assessed viability by measuring cellular ATP (Fig. S4B). Neither 4μ8C nor STF-083010 was cytotoxic alone. In contrast to our prediction, we found that neither IRE1α inhibitor sensitized HCoV-OC43-infected cells to die. These data suggest that this virus does not require IRE1α to maintain host cell viability and uses IRE1α for another aspect of its life cycle.

### XBP1 is required for optimal HCoV-OC43 infection

**Fig 4:**
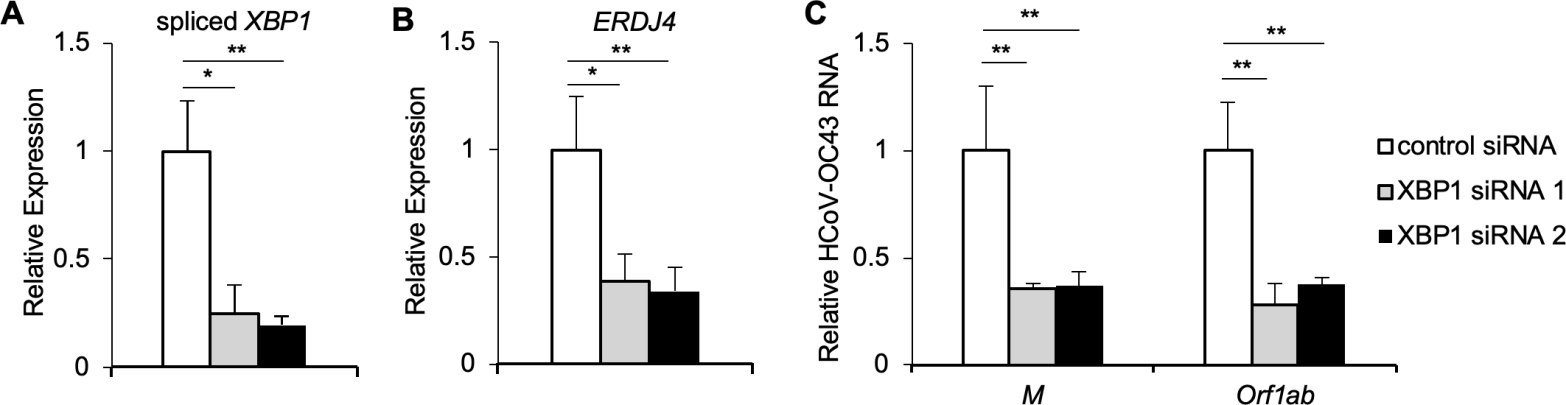
XBP1 is required for optimal HCoV-OC43 infection. HCT-8 cells were transfected with siRNA targeting XBP1 or non-targeting control siRNA and then infected with HCoV-OC43. RNA was harvested 48-hour post-infection, and the relative abundance of spliced *XBP1* (**A**), *ERDJ4* (**B**), and HCoV-OC43 viral RNA (**C**) was determined by quantitative RT-PCR. Data are means ± SD of six replicates and are representative of three independent experiments. *P < 0.01, **P < 0.005 by unpaired *t*-test.

The inhibitors 4μ8c and STF-083010 are selective for the IRE1α nuclease domain and have no effect on the kinase activity of IRE1α (27). Our results demonstrate XBP1 target induction during HCoV-OC43 infection (Fig. 1B and C), suggesting that the requirement for IRE1α could be via *XBP1* splicing and transcriptional induction of XBP1 targets. To test the hypothesis that IRE1α promotes HCoV-OC43 infection via XBP1, we knocked down XBP1 using siRNA. We found a significant reduction in spliced *XBP1* mRNA (Fig. 4A), sXBP1 protein (Fig. S2A and B), and expression of the XBP1-induced target *ERDJ4* (Fig. 4B) in cells treated with two independent siRNAs targeting XBP1. We additionally found that knocking down *XBP1* reduced HCoV-OC43 RNA abundance (Fig. 4C), indicating that the requirement for IRE1α in HCoV-OC43 infection is XBP1-dependent.

### IRE1α and XBP1 promote SARS-CoV-2 infection and inflammatory cytokine responses independently of viral entry

**Fig 5:**
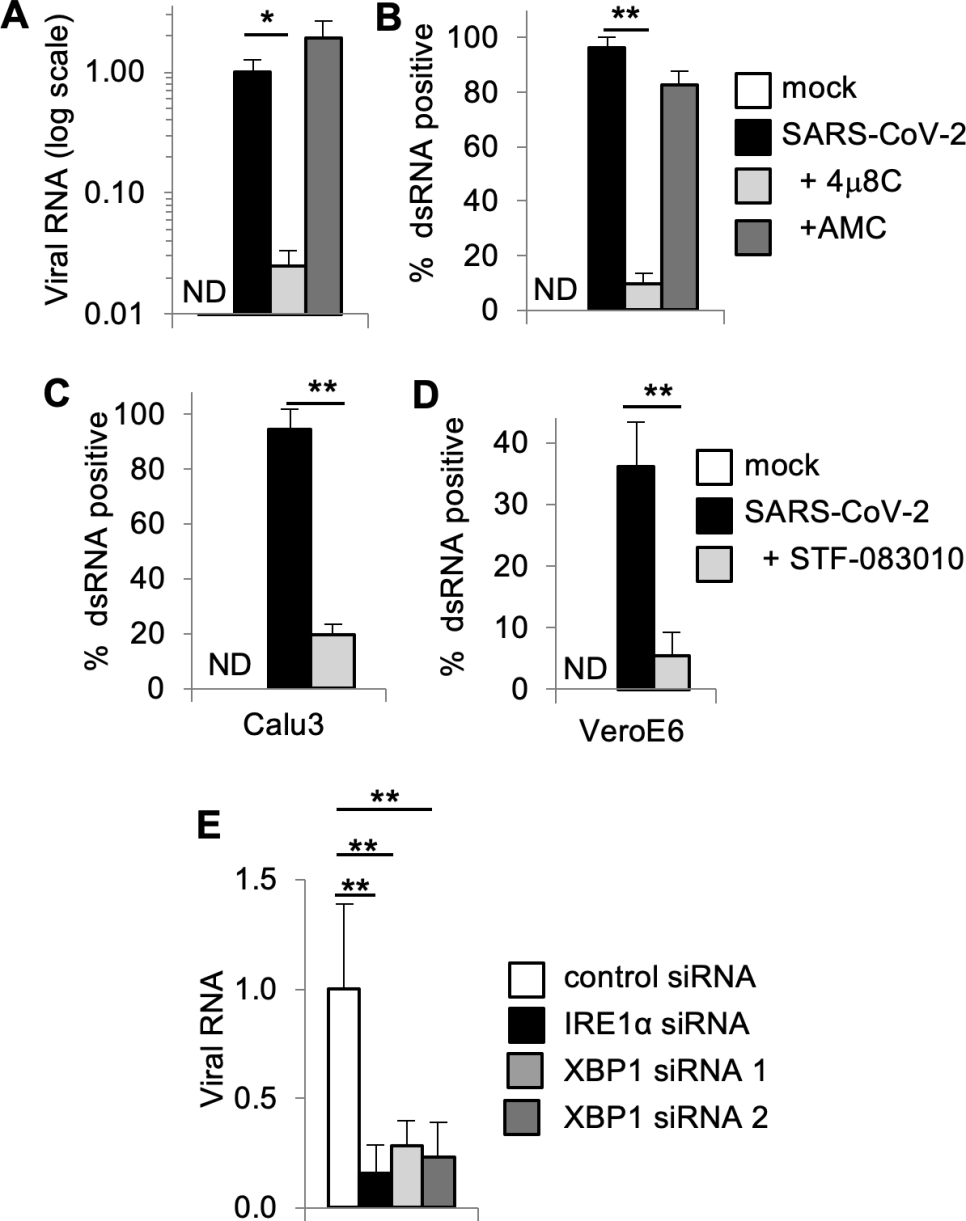
IRE1α and XBP1 are required for optimal SARS-CoV-2 infection. (A+**B**) Calu-3 cells were treated with IRE1α nuclease inhibitor 4μ8C, structurally similar negative control AMC or DMSO solvent control prior to infection with SARS-CoV-2. (**A**) RNA was harvested 48-hour post-infection, and the relative abundance of viral RNA was determined by quantitative RT-PCR. (**B**) The viral replication intermediate, dsRNA, was visualized by immunostaining and positive cells were quantified. (**C**) Calu-3 or (**D**) VeroE6 cells were treated with IRE1α nuclease inhibitor STF-083010 or DMSO solvent control prior to infection with SARS-CoV-2 and dsRNA immunostaining. (**E**) Calu-3 cells were transfected with siRNA targeting IRE1α, XBP1, or non-targeting control siRNA prior to SARS-CoV-2 infection and measurement of viral RNA by quantitative RT-PCR. Data are means ± SD of three (**A, B, C**), five (**D**), or six (**E**) replicates and are representative of two independent experiments. *P < 0.05, **P < 0.01 by unpaired *t*-test. ND, not detected.

In addition to HCoV-OC43, we found that SARS-CoV-2 stimulates robust IRE1α activation with *XBP1* splicing and induction of XBP1 target genes (Fig. 1D and E). To determine whether the requirement for IRE1α is shared by this highly pathogenic human coronavirus, we tested the IRE1α nuclease inhibitor, 4μ8C. Inhibiting IRE1α with 4μ8C reduced the abundance of viral RNA by almost 2-logs at 48-hour post-infection, whereas the inactive control had no effect (Fig. 5A). Similarly, to our results with HCoV-OC43, we found that the structurally distinct IRE1α nuclease inhibitor, STF-083010 also reduced viral RNA at this time point, but viral RNA was unaffected by either inhibitor at an earlier time point (Fig. S5A) in a low MOI infection model. To determine whether IRE1α also supports viral replication during a high MOI infection, we infected cells with an MOI of 1. We again observed a significant reduction of viral RNA in cells treated with the IRE1α nuclease inhibitor, 4μ8C, but not inactive control AMC (Fig. S5B). Together, these results indicate that efficient SARS-CoV-2 replication requires the activity of this host protein.

To verify these results, we performed immunostaining for dsRNA and observed that 4μ8C, but not the inactive control, robustly inhibited viral infection (Fig. 5B). The structurally distinct IRE1α nuclease inhibitor STF-083010 also strongly limited viral infection as assessed by dsRNA immunostaining (Fig. 5C). Neither 4μ8C nor STF-083010 were cytotoxic to Calu-3 cells, nor did they reduce the viability of SARS-CoV-2-infected cells (Fig. S5C). To determine whether this effect would extend to other cell types, we infected VeroE6 cells and confirmed the inhibition of SARS-CoV-2 infection with the IRE1α inhibitor, STF-083010 (Fig. 5D) without an effect on cell viability (Fig. S5D).

To test the hypothesis that IRE1α promotes SARS-CoV-2 infection via XBP1, we knocked down XBP1 using siRNA in Calu-3 cells. We verified the reduction in spliced *XBP1* mRNA with XBP1 siRNA (Fig. S5E). We found a significant reduction in SARS-CoV-2 viral RNA in cells treated with two independent siRNAs targeting *XBP1* (Fig. 5E). We also genetically confirmed the requirement for IRE1α, as IRE1α siRNA knockdown also suppressed viral replication (Fig. 5E). Together, these data demonstrate that SARS-CoV-2 requires host IRE1α RNase activity and XBP1 for optimal infection.

**Fig 6:**
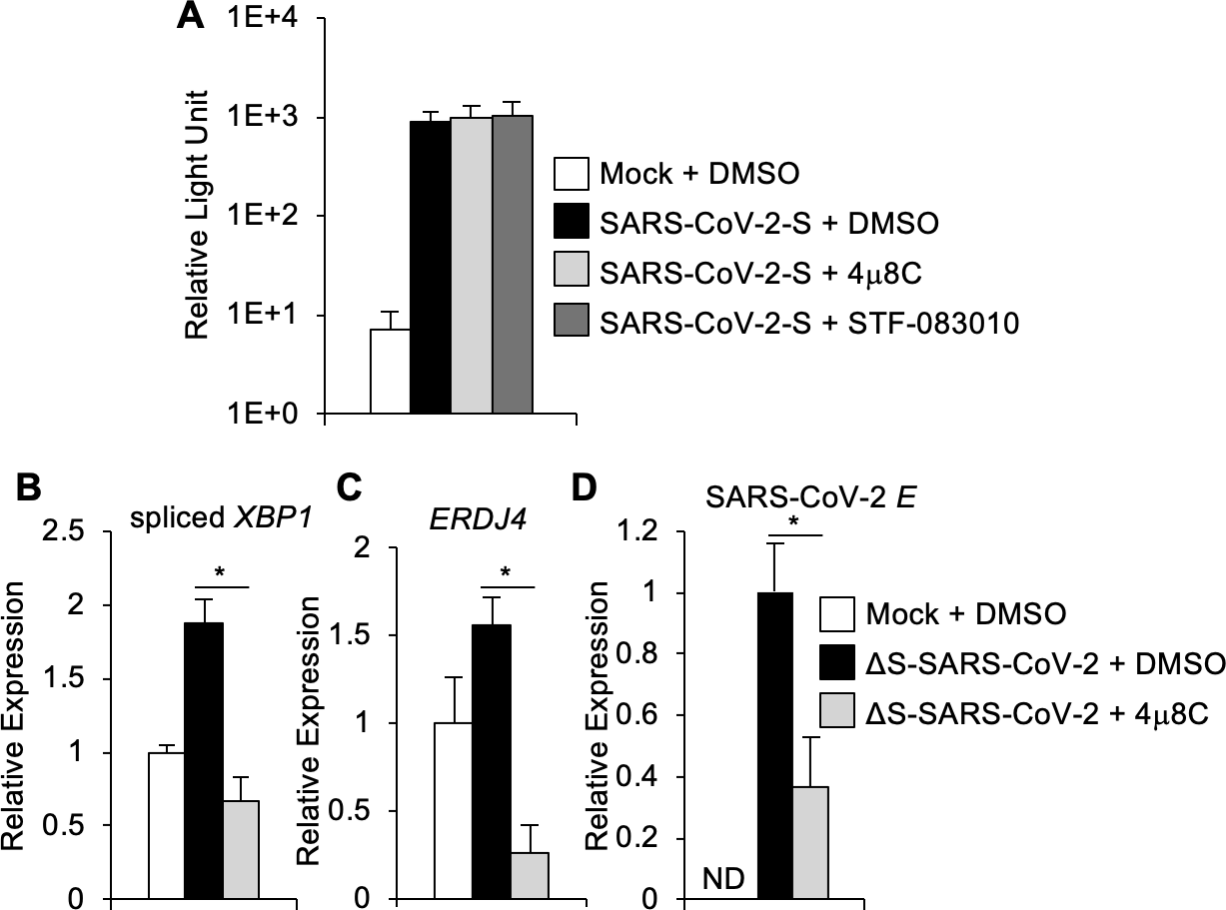
IRE1α promotes SARS-CoV-2 viral RNA replication independently of viral entry. (**A**) Hek293 + ACE2 cells were pre-treated with small molecule IRE1α inhibitors or DMSO solvent control prior to infection with SARS-CoV-2 spike pseudotyped lentivirus or mock infection. Reporter virus infection was assessed 72 hours later by measuring luciferase activity, which is shown on a log scale. (**B–D**) Calu-3 cells were treated with IRE1α nuclease inhibitor 4μ8C or DMSO solvent control prior to infection with ΔS-SARS-CoV-2 single-cycle virus replicon particles. RNA was harvested 24 hours post-infection, and the relative abundance of spliced *XBP1* (**B**), *ERDJ4* (**C**), or SARS-CoV-2 E gene (**D**) was determined by quantitative RT-PCR. Data are means ± SD of eight (**A**) or five (**B**) replicates and are representative of two (**A**) or three (**B–D**) independent experiments, respectively. *P < 0.05 by unpaired *t*-test. ND, not detected.

Coronaviruses, including SARS-CoV-2, interact with cell surface receptors via their spike envelope glycoprotein to initiate receptor-mediated entry. To determine whether IRE1α supports the initial viral binding and entry processes, we used SARS-CoV-2 spike pseudotyped lentivirus carrying a luciferase reporter (28). We infected HEK293T cells by overexpressing the ACE2 receptor and assessed reporter virus infection by measuring luciferase activity. We found that the IRE1α inhibitors 4μ8C and STF-083010 did not prevent pseudovirus infection (Fig. 6A). Together with our earlier observation that IRE1α inhibitors had no significant effect on viral RNA abundance at an early time point post-infection, these results suggest that receptor binding and spike-dependent entry are not IRE1α-dependent.

To further examine post-entry aspects of viral replication, we used a single-cycle infectious SARS-CoV-2 virus replicon particle system (29). This approach utilizes co-transfection of a bacterial artificial chromosome–encoded viral genome in which spike is deleted, together with plasmid-encoded VSV-G. Infection with the resulting virus replicon particles is mediated by VSV-G and independent of ACE2. Infection is limited to a single cycle, as viral RNA replication, but not subsequent virion packaging, can occur. We infected cells with single-cycle virus replicon particles and found that 4μ8C prevented *XBP1* mRNA splicing (Fig. 6B) and subsequent *ERDJ4* induction (Fig. 6C) verifying its effect on IRE1α. We observed that 4μ8C significantly reduced viral RNA in this system (Fig. 6D). Together, these data suggest that IRE1α supports post-entry SARS-CoV-2 viral RNA replication.

### ER stress enhances human coronavirus infection via IRE1α

**Fig 7:**
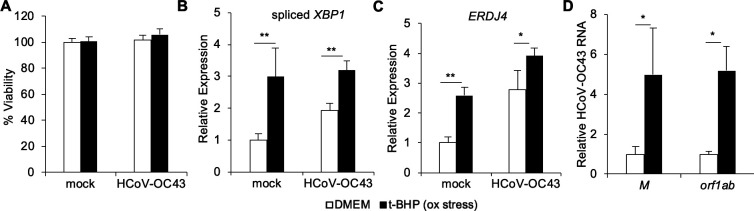
Pre-treatment with *tert-*butyl hydroperoxide enhances HCoV-OC43 viral infection. HCT-8 cells were treated with the oxidative stress inducer, *tert*-butyl hydroperoxide (t-BHP) or medium control 2 hours prior to infection with HCoV-OC43. Viability was measured by quantifying dehydrogenase activity in metabolically active cells (**A**). RNA was harvested 48 hours post-infection, and the relative abundance of spliced *XBP1* (**B**), *ERDJ4* (**C**), and HCoV-OC43 viral RNA (**D**) was determined by quantitative RT-PCR. Data are means ± SD of four replicates and are representative of two independent experiments.*P < 0.05, **P < 0.001 by unpaired *t*-test.

Risk factors for severe COVID-19 include advanced age, hypertension, diabetes, and obesity, which are all associated with ER stress (8 - 30 - 31 - 31). Although there are likely multiple mechanisms contributing to disease severity, we hypothesized that conditions associated with pre-existing ER stress may prime exuberant viral replication via IRE1α. Oxidative stress is associated with aging and metabolic diseases, causes ER stress, and activates IRE1α (32 - 34). To determine if exogenous oxidative stress enhances coronavirus infection, we pre-treated cells with the oxidative stress inducer tBHP (35, 36) at non-cytotoxic concentrations (Fig. 7A). We verified that tBHP triggered *XBP1* splicing (Fig. 7B) and expression of the XBP1 target gene *ERDJ4* (Fig. 7C), consistent with prior studies (36). Oxidative stress induced by tBHP enhanced HCoV-OC43 infection, as indicated by increased viral RNA (Fig. 7D). To understand whether the effects on increased viral RNA were due to IRE1α activity, we pre-treated cells with tBHP paired with IRE1α nuclease inhibitor prior to HCoV-OC43 infection. We found that cells treated with tBHP and either 4μ8C or STF-083010 had viral RNA levels (Fig. S6A) and *XBP1* splicing (Fig. S6B) similar to DMSO-only control samples. These results overall indicate that oxidative stress activates IRE1α and promotes HCoV-OC43 infection.

### XBP1 activation occurs during human COVID-19

**Fig 8:**
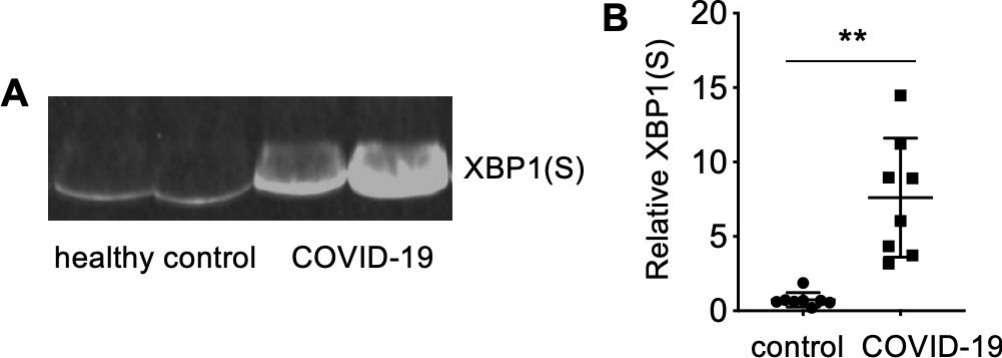
Elevated serum XBP1(S) in human SARS-CoV-2 infection. (A+B) XBP1(S) was measured in serum from normal healthy controls or patients with COVID- 19 by western blot. (**A**) The image shows representative samples. (**B**) Relative quantification. Means + SD, *n* = 8. **P < 0.001 by Mann–Whitney *U* test.

We hypothesized that SARS-CoV-2 infection activates IRE1α not only in cultured cells but also in patients with COVID-19 and may represent a prognostic marker for disease severity. To test this hypothesis, we measured the protein product of spliced XBP1 in serum from patients with severe COVID-19 and normal healthy controls using western blot (Fig. 8A). We found a strong and consistent increase in XBP1(S) protein in samples from patients hospitalized in the intensive care unit with acute, severe COVID-19 compared to normal controls (Fig. 8B). This finding suggests that XBP1 activation occurs not only in cultured cells but also in humans suffering from severe SARS-CoV-2 infection.

## DISCUSSION

In this study, we found that HCoV-OC43 and SARS-CoV-2 both robustly activate the IRE1α–XBP1 branch of the unfolded protein response in cultured cells. The abundance of the protein product of spliced XBP1 in patients with COVID-19 indicates activation of this pathway also occurs during human infection. Although we focused on the IRE1α–XBP1 branch of the unfolded protein response, Protein kinase RNA (PKR)-like ER kinase (PERK) and activating transcription factor-6 (ATF6) have also been found to be activated during infection with SARS-CoV-2, but interestingly not HCoV-OC43 (16, 19, 37, 38). These responses may be influenced by cell type and infection conditions, as partial IRE1α activation was found in SARS-CoV-2-infected A549 cells and during infection with a higher MOI of 5 (39). Compared to other cell types, A549 cells also demonstrate reduced *XBP1* splicing in response to the chemical ER stress inducers thapsigargin and tunicamycin (40). SARS-coronavirus limits IRE1α activation via the envelope (E) gene (18). The E gene is conserved, but not identical between SARS-CoV and SARS-CoV-2 (41), and there are multiple other genetic differences between these two viruses, which contribute to differences in biological behavior via poorly understood mechanisms. Future studies may reveal specific determinants of differential IRE1α regulation between human coronaviruses and during different infection conditions.

There may be multiple pathways contributing to IRE1α activation during HCoV-OC43 and SARS-CoV-2 infection. For example, overexpression of either SARS-CoV-2 ORF3a, ORF8, or spike proteins is sufficient to activate all three branches of the unfolded protein response, including IRE1α activation and *XBP1* mRNA splicing (16, 37, 42, 43). In addition, SARS-CoV-2 NSP6 interacts with the sigma-1 receptor (44), an ER-resident transmembrane protein that contributes to IRE1α activation (45). Although *XBP1* splicing occurs during infection, we found no evidence of degradation of other IRE1α targets via RIDD in HCoV-OC43- or SARS-CoV-2-infected cells. *XBP1* splicing and RIDD are distinct outputs of the IRE1α RNase activity that are not always present simultaneously (46) and may be differentially regulated by the oligomerization state of IRE1α (47). Thus, viral infection may be sufficient to trigger *XBP1* splicing, but limit broader RNA degradation.

COVID-19 is characterized by a curiously broad spectrum of disease severity, with some patients having mild to no symptoms and others succumbing to lethal disease (48, 49). Risk factors for severe disease include advanced age, diabetes, hypertension, obesity, and increased viral load (50, 51). HCoV-OC43 is also associated with a spectrum of clinical symptoms and has been associated with lethal infections (52). Although the mechanisms contributing to severe coronavirus disease are likely multifactorial, all of these risk factors are well-associated with ER stress and IRE1α activation (2).

IRE1α promotes infection by a number of different viruses via distinct mechanisms. XBP1-independent activities of IRE1α limit apoptosis of cells infected with the avian coronavirus (IBV) (12), as well as hepatitis C virus (13). Unlike these studies, we found that IRE1α inhibition did not result in the death of HCoV-OC43- or SARS-CoV-2-infected cells, suggesting that IRE1α does not play an anti-apoptotic role for these viruses. IRE1α promotes replication of the porcine coronavirus TGEV via XBP1-independent evasion of antiviral interferon responses (11). We found the activity of IRE1α inhibition against SARS-CoV-2 in interferon-deficient (53) VeroE6 cells, and a requirement for XBP1, suggesting a mechanism distinct from interferon evasion. IRE1α inhibition had no effect on SARS-CoV-2 spike pseudotyped viral entry, suggesting that receptor binding and spike-dependent entry are not IRE1α-dependent. Instead, we hypothesize that IRE1α leads to XBP1-dependent transcriptional changes that promote post-entry aspects of viral replication. In support of this, we found that IRE1α supports optimal viral RNA replication in a single-cycle ACE2-independent SARS-CoV-2 virus replicon particle system. Future work is necessary to further define the specific step(s) of the viral life cycle that are supported by IRE1α and determine if this host factor impacts initial translation, polyprotein processing, or viral RNA replication. Spliced XBP1 is required and sufficient to expand the ER in specialized secretory cells and induce morphological alterations including perinuclear vesicles that appear similar to changes observed with SARS-CoV-2 infection (6, 54 - 58). These observations suggest the hypothesis that IRE1α may support the biogenesis of ER-derived viral replication platforms. Further work is needed to understand whether IRE1α differentially affects specific aspects of viral RNA replication, including synthesis of negative RNA or positive genomic or subgenomic RNA species. Overall, this warrants future study.

In addition to regulating ER-associated genes, XBP1 binds directly to promoters for cytokines including interleukin 6 (IL-6) (59), which is strongly induced in severe COVID-19 and associated with the risk of respiratory failure and death (60 - 62). However, coronaviruses disrupt many pathways of pattern recognition receptor-mediated innate immune sensing (63), and XBP1-mediated cytokine production could provide a means of inciting inflammation via the detection of pathogen-induced cellular stress, even in the setting of viral innate immune antagonism. In addition to promoting acute inflammation, IRE1α is associated with chronic lung injury and pulmonary fibrosis (64, 65). We are only beginning to understand the long-term consequences faced by patients after acute COVID-19, which include symptoms from pulmonary fibrosis (66, 67) that may be a consequence of viral IRE1α activation. Based on emerging evidence for the role of IRE1α in multiple diseases, IRE1α inhibitors have been developed and evaluated as potential therapeutics. These drugs have provided robust *in vivo* inhibition of IRE1α and have been well-tolerated in both pre-clinical studies and early clinical trials in humans (27, 68 - 70). Ultimately, based on the cellular findings presented here, further animal studies are warranted to determine the impact of IRE1α on viral pathogenesis and replication *in vivo*.

## MATERIALS AND METHODS

### Reagents

Cells were treated with 1 µg/mL tunicamycin (Sigma-Aldrich), 50 µM 4μ8c (8-formyl-7-hydroxy-4-methylcoumarin, MilliporeSigma), 50 µM AMC (7-amino-4-methylcoumarin, VWR), 50 µM STF-083010 (MilliporeSigma), or 50 µM tBHP (*tert*-butyl hydroperoxide; Sigma-Aldrich). Viability of HCoV-OC43 infected cells was assessed using the CellTiter 96 AQueous One Solution Cell Proliferation Assay (Promega). Viability of SARS-CoV-2-infected cells was assessed using the CellTiter-Glo 2.0 Assay (Promega).

### Cells and viruses

Cells were propagated in Dulbecco’s modified Eagle’s medium (DMEM) (Gibco) supplemented with 10% fetal bovine serum (FBS) (Serum Plus II, MilliporeSigma), 10 mM HEPES, 50 U/mL penicillin-streptomycin (Gibco), and 0.05 mM β-mercaptoethanol. HCT-8, Calu-3, and VeroE6 cells were obtained from the American Type Culture Collection. 293T-ACE2 cells that overexpress ACE2 (28) were a kind gift from Dr. Jesse D. Bloom (Fred Hutchinson Cancer Research Center, Seattle, WA, USA). HCoV-OC43 (NR-52725) was obtained through BEI Resources, National Institute of Allergy and Infectious Diseases (NIAID), National Institutes of Health (NIH) and propagated in HCT-8 cells using medium containing 2% FBS. HCoV-OC43 was titered in HCT-8 cells using a focus-forming assay and used for infections at an MOI of 0.01, unless otherwise indicated. SARS-CoV-2 Isolate USA-WA1/2020 (NR-52281) was obtained through BEI Resources, NIAID, NIH; propagated in VeroE6 cells; titered in VeroE6 cells using a plaque-forming assay; and used for infections at an MOI of 0.01, unless otherwise indicated. Cells were treated with small-molecule IRE1α inhibitors for 2.5 hours prior or tBHP for 2 hours prior to infection.

### Single-cycle infectious SARS-CoV-2 virus replicon particles

The generation of SARS-CoV-2 virus replicon particles (VRP(G)) was performed using the delta spike dual reporter bacmid and vesicular stomatitis virus glycoprotein G (VSV-G) plasmid (Addgene; 138479) as described previously (29) with some modifications. Briefly, a mixture of 293T/Huh7.5 cells (−1 × 10^6^ cells of each type) was transfected with 3.5 µg of ΔS-Luc-GFP (green fluorescent protein) bacmid and 0.5 µg of VSV-G plasmid using polyethyleneimine (PEI) transfection reagent (Polysciences; DNA/PEI ratio, 1:4). At 5–6 hours, the transfection mixture containing media was replaced with DMEM/2% and incubated at 37°C. At 48–72 hours post media change, the supernatants were collected and used seed stocks for producing ΔS-VRP(G) working stocks. To generate working stocks, approximately 1.5–2 × 10^7^ Huh7.5 cells seeded in 15-cm plates a day prior were transfected with 20 μg of VSV-G plasmid using PEI reagent (DNA/PEI ratio, 1:4), and the media was changed to DMEM/10% FBS at 5–6 hours post-transfection. The following day, VSV-G-transfected Huh7.5 cells were placed in 10-mL DMEM/2% FBS and infected with 1 mL of ΔS-VRP(G) seed stock. After 2–3 hours of incubation, the inoculum mixture was replaced with 25 mL of DMEM/2%FBS and GFP expression in the cells as well as the luciferase activity in the supernatant. At the peak of GFP expression or luciferase activity (~24–48 hours post media change), the supernatants were collected, clarified of cellular debris by centrifugation at 3,000 rpm for 10 minutes, and stored at –80°C until use. Calu-3 cells were seeded in 96-well plates and treated with small molecule IRE1α inhibitor 4u8C or DMSO for 2.5 hours. Cells were then incubated for 3 hours with 50 μL of ΔS-VRP(G) seed stock, and plates were rocked back and forth every 15 minutes during the incubation period. The supernatant was removed and replaced with a fresh medium containing IRE1α inhibitor or DMSO.

### Expression analysis

RNA isolated from cell lysates using the SingleShot Cell Lysis Kit (Bio-Rad) was used to synthesize cDNA using the iScript cDNA Synthesis Kit (Bio-Rad). Quantitative RT-PCR (qRT-PCR) was performed on a Bio-Rad CFX Connect or Roche Lightcycler 480 using SYBR Green (Bio-Rad) with the following primers (all primers listed in the 5′–3′ orientation):

human spliced *XBP1*: TGCTGAGTCCGCAGCAGGTG (forward) and GCTGGCAGGCTCTGGGGAAG (reverse)

human *ERDJ4*: TAGTCGGAGGGTGCAGGATA (forward) and CGCTCTGATGCCGATTTTGG (reverse)

human *P58IPK*: (forward) TGTGTTTGGGATGCAGAACTAC and (reverse) TCTTCAACTTTGACGCAGCTT

human *BLOC1S1*: (forward) CAGACAGGCCAGTGGATCG and (reverse) TCTCCACATCCCCAATTTCCTTG

human *SCARA3*: (forward) GTGTTGGCCTCTCTGGTTTTC and (reverse) AAGAGCAGTTGTTCAGGGCT

human *COL6A1*: (forward) ATGTGCTCTTGCTGTGAATGC and (reverse) GAAGTTCTGCAGGCCAATGC

human *HGSNAT*: (forward) CACCTTCAGGGGGATTGCTC and (reverse) TACAAACCACGGGAACACGA

human *RPS18*: TGC GAG TAC TCA ACA CCA ACA (forward) and CTT CGG CCC ACA CCC TTA AT (reverse)

human *GAPDH*: CAA TGA CCC CTT CAT TGA CC (forward) and GAC AAG CTT CCC GTT CTC AG (reverse)

HCoV-OC43 *M*: ATG TTA GGC CGA TAA TTG AGG ACT AT (forward) and AAT GTA AAG ATG GCC GCG TATT (reverse)

HCoV-OC43 orf1ab: TGG ATT TTG GCG GGA TGG AA (forward) and GAG ACG GGC ATC TAC ACT CG (reverse)

SARS-CoV-2 E: ACAGGTACGTTAATAGTTAATAGCGT and ATATTGCAGCAGTACGCACACA

Melt curve analysis was used to assess whether single reaction products were produced. Expression was calculated relative to the housekeeping gene *RPS18*, with equivalent results also obtained relative to *GAPDH*.

### siRNA

HCT-8 or Calu-3 cells were transfected with siRNA against IRE1α (D-004951-01-0005, Dharmacon), XBP1 (D-009552-02-005, D-009552-0005, Dharmacon), or no-target control siRNA (D-001210-03-05, Dharmacon) using Lipofectamine RNAiMax Transfection Reagent (Invitrogen) according to the manufacturer’s instructions.

### Immunofluorescence microscopy

After fixation in 2% paraformaldehyde, cells were permeabilized with 0.2% Triton-X100 in phosphate-buffered saline (PBS) and blocked with 3% bovine serum albumin + 0.2% Triton-X100 in PBS. Cells were labeled with anti-dsRNA mouse monoclonal antibody J2 (SCICONS, catalog no. 10010200) and Alexa Fluor 488 secondary antibody (Invitrogen, catalog no. A-21202). TO-PRO-3 (Invitrogen, catalog no. T3605) was used to label nuclei. For HCoV-OC43 infected cells, a Cytation 1 fluorescent cell imaging system (BioTek) was used for image acquisition (10× objective), and Gen5 software (BioTek) was used for image processing and subsequent analysis. For dsRNA staining, total fluorescence intensity was measured with four wells per condition using identical capture settings for the target of interest. The total fluorescence intensity from the uninfected DMSO-negative control was subtracted from all condition wells. For SARS-CoV-2-infected cells, images were collected with an EVOS FLoid cell imaging station (ThermoFisher). For quantification of dsRNA staining of SARS-CoV-2-infected cells, the total number of cells was determined by counting the number of nuclei, and the percentage of dsRNA-positive cells was calculated using this and the number of cells with positive dsRNA staining.

### Coronavirus spike-mediated pseudovirus entry assay

Lentivirus pseudotyped with SARS-CoV-2 spike was generated in HEK293T cells, and pseudovirus entry assay was performed as previously described (28). Plasmids expressing the HIV-1 Gag and pol (pHDM-Hgpm2), HIV-1 Rev (pRC-CMV-rev1b), HIV-1 Tat (pHDM-tat1b), C-terminally truncated SARS CoV2 spike (pHDM-SARS-CoV-2delta21 Spike), and luciferase/GFP reporter (pHAGE-CMV-Luc2-IRES-ZsGreen-W) were co-transfected into HEK293T cells using Lipofectamine 3000 (ThermoFisher) according to the manufacturer’s instructions. Plasmids were a kind gift from Dr. Jesse D. Bloom (Fred Hutchinson Cancer Research Center, Seattle, WA, USA). Pseudovirus was harvested 60 hours post-transfection and stored at −80°C. 293T-ACE2 cells were pre-treated with IRE1α inhibitors for 2.5 hours prior to pseudovirus infection. Luciferase reporter gene expression was assessed 72 hours post-infection by quantifying relative luminescence units using the ONE-GLO Luciferase Assay System (Promega).

### Western blot

Protein extraction from cultured cells was performed with Protein Extraction Reagent Type 4 (MilliporeSigma) with added HALT protease Inhibitor Cocktail (ThermoFisher), phenylmethylsulfonyl fluoride (PMSF) protease inhibitor (ThermoFisher), and Benzonase nuclease (MilliporeSigma), mixed with loading buffer and heated at 95°C for 10 minutes under reducing conditions. Serum specimens from patients with RT-PCR-confirmed SARS-CoV-2 infection were collected from the clinical laboratories at the University of Washington and Harborview Medical Centers in Seattle, WA, USA, between March and May of 2020 (71). Specimens were collected under an institutional review board-approved waiver of consent from patients receiving care in the intensive care unit. Samples from healthy blood donors obtained prior to the COVID-19 pandemic were used as normal controls. Specimens were stored at −80°C before testing. Thawed specimens were diluted 1:30 with digestion buffer Protein Extraction Reagent Type 4 (MilliporeSigma) with added HALT protease Inhibitor Cocktail (ThermoFisher), PMSF protease inhibitor (ThermoFisher), and benzonase nuclease (MilliporeSigma), mixed with loading buffer and heated at 95°C for 10 minutes under reducing conditions. Proteins were separated by SDS-PAGE using Any kD TGX stain-free gels (Bio-Rad) and transferred to nitrocellulose membranes. Membranes were probed with rabbit polyclonal anti-XBP1 (Invitrogen, catalog no. PA5-27650) or rabbit monoclonal anti-XBP1 (Cell Signaling Technology, catalog no. D2C1F) and mouse monoclonal anti-vinculin (Santa Cruz Biotechnology, catalog no. sc-73614) antibodies, followed by incubation with secondary antibody, donkey anti-rabbit IRDye 800CW (LI-COR, catalog no. 926-32213), and goat anti-mouse IRDye 680RD (LI-COR, catalog no. 926-68073). The blots were imaged with an Odyssey Infrared Imaging System (LI-COR Biosciences) and relative density units were calculated with Image Studio Lite Version.

### Statistics

The unpaired two-tailed Student’s *t*-test or the Mann–Whitney *U* test was used for comparisons between the two groups. *P* values of less than 0.05 were considered statistically significant.

## SUPPLEMENTAL MATERIAL

Figure S1 mbio.00540-23-s0001.pdf Regulated IRE1α-dependent decay.

Figure S2 mbio.00540-23-s0002.pdf sXBP1 protein.

Figure S3 mbio.00540-23-s0003.pdf IRE1α is required for optimal HCoV-OC43.

Figure S4 mbio.00540-23-s0004.pdf IRE1α inhibitors reduce viral protein and are not cytotoxic.

Figure S5 mbio.00540-23-s0005.pdf IRE1α inhibitors decrease SARS-CoV-2.

Figure S6 mbio.00540-23-s0006.pdf Pretreatment with tert-butyl hydroperoxide.

ASM does not own the copyrights to Supplemental Material that may be linked to, or accessed through, an article. The authors have granted ASM a non-exclusive, world-wide license to publish the Supplemental Material files. Please contact the corresponding author directly for reuse.
