# Supplementary material for: The unfolded protein response components IRE1α and XBP1 promote human coronavirus infection
Source: mBio. 2023 Jun 12;14(4):e00540-23. doi: 10.1128/mbio.00540-23 (PMC10470493; doi:10.1128/mbio.00540-23)
Supplement: Figure S1 — Regulated IRE1α-dependent decay. [file mbio.00540-23-s0001.pdf]

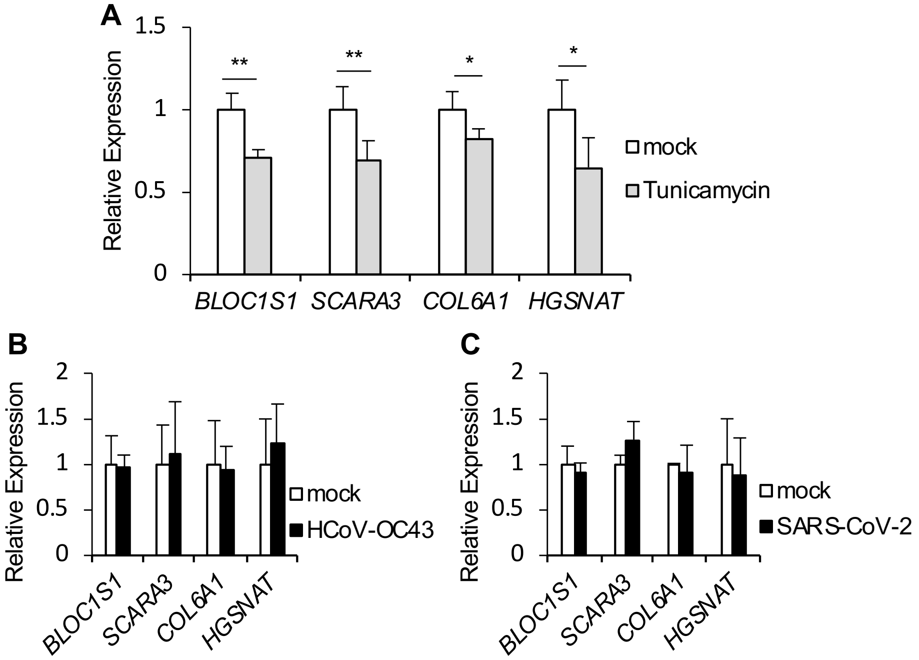

Supplemental Figure 1. **Regulated IRE1α-dependent decay (RIDD) is not activated by HCoV-OC43 or SARS-CoV-2 infection. (A-C)** Quantitative RT-PCR was used to determine the relative abundance of RIDD-targets *BLOC1S1, SCARA3, COL6A1,* and *HGSNAT* in RNA from **(A)** HCT-8 cells treated with the ER stress-inducing agent, tunicamycin for 5 hours, **(B)** HCT-8 cells infected with HCoV-OC43 for 48 hours, or **(C)** Calu-3 cells infected with SARS-CoV-2 for 48 hours. Data are means ± SD of four replicates and are representative of two independent experiments. *$P < 0.05$, ** $P < 0.01$, by unpaired *t* test.
