# Supplementary material for: The unfolded protein response components IRE1α and XBP1 promote human coronavirus infection
Source: mBio. 2023 Jun 12;14(4):e00540-23. doi: 10.1128/mbio.00540-23 (PMC10470493; doi:10.1128/mbio.00540-23)
Supplement: Figure S2 — sXBP1 protein. [file mbio.00540-23-s0002.pdf]

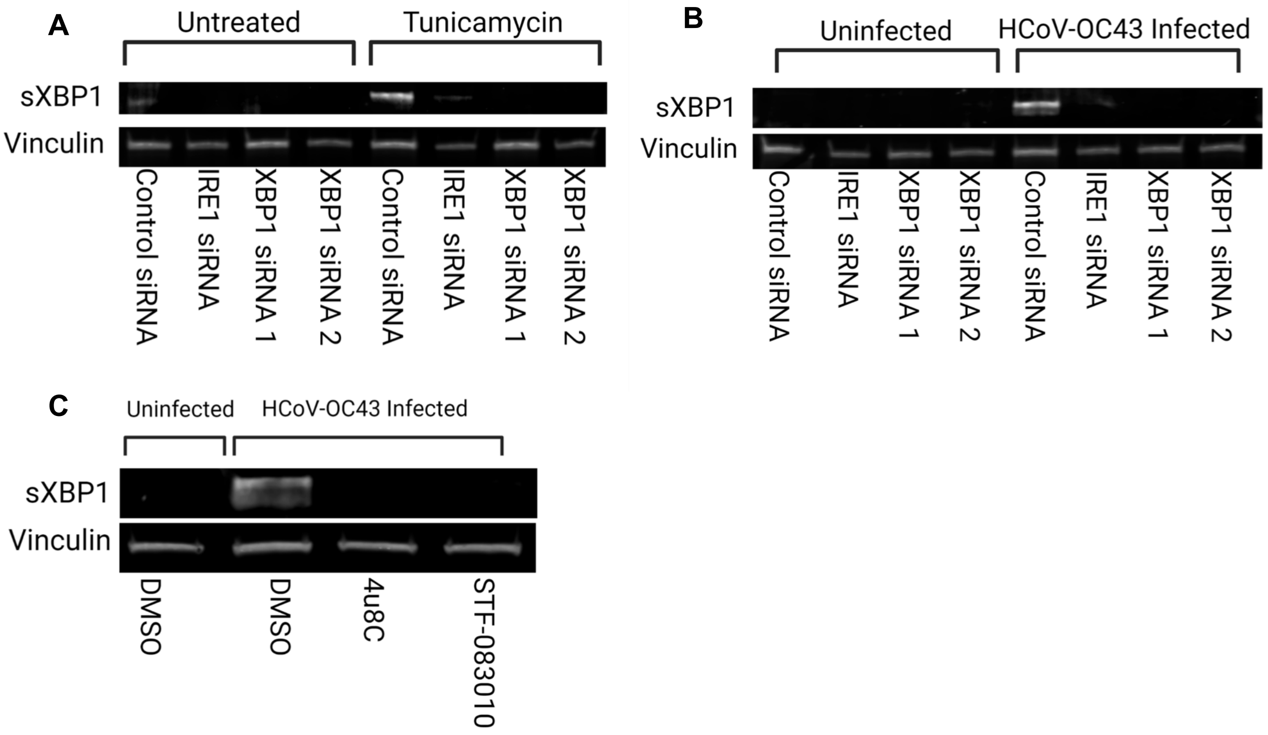

Supplemental Figure 2. **IRE1α small molecule inhibitors and transfection of XBP1 and IREα siRNA effectively knock down sXBP1 protein. (A+B)** HCT-8 cells were transfected with siRNA targeting XBP1, IRE1α, or non-targeting control siRNA and then treated with tunicamycin **(A)** or infected with HCoV-OC43 **(B)**. **(C)** HCT-8 cells were pretreated with DMSO or IRE1α small molecule inhibitors and then infected with HCoV-OC43. Protein was harvested 5 hours after tunicamycin treatment **(A)** or 48 hours after HCoV-OC43 infection **(B+C)**. The abundance of sXBP1 protein and vinculin loading control were evaluated by Western blot. Data are representative of two independent experiments.
