# Supplementary material for: The unfolded protein response components IRE1α and XBP1 promote human coronavirus infection
Source: mBio. 2023 Jun 12;14(4):e00540-23. doi: 10.1128/mbio.00540-23 (PMC10470493; doi:10.1128/mbio.00540-23)
Supplement: Figure S3 — IRE1α is required for optimal HCoV-OC43. [file mbio.00540-23-s0003.pdf]

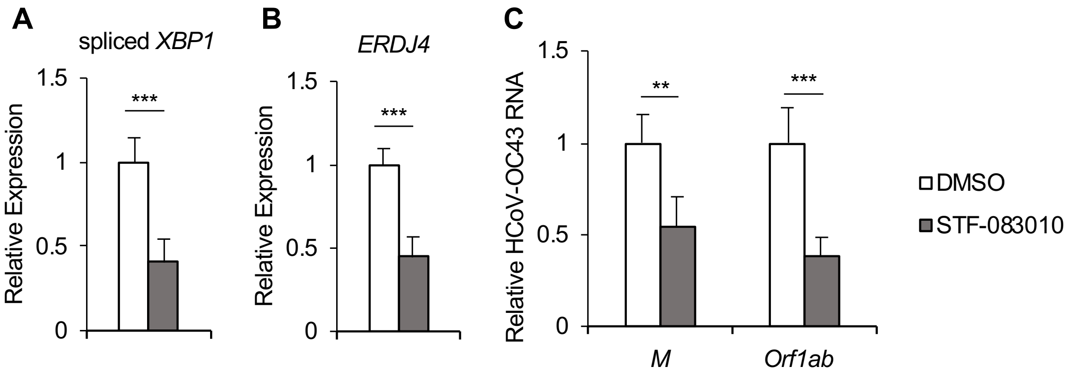

Supplemental Figure 3. **IRE1α is required for optimal HCoV-OC43 infection. (A-C)** HCT-8 cells were treated with IRE1α nuclease inhibitor STF-083010 or DMSO solvent control prior to infection with HCoV-OC43. RNA was harvested 48 hours post-infection and the relative abundance of spliced *XBP1* **(A),** *ERDJ4* **(B),** and HCoV-OC43 viral RNA **(C)** were determined by quantitative RT-PCR. Data are means ± SD of four replicates and are representative of three independent experiments. $^{*}P < 0.05$, $^{**}P < 0.01$, $^{***}P < 0.001$, by unpaired *t* test.
