# Supplementary material for: The unfolded protein response components IRE1α and XBP1 promote human coronavirus infection
Source: mBio. 2023 Jun 12;14(4):e00540-23. doi: 10.1128/mbio.00540-23 (PMC10470493; doi:10.1128/mbio.00540-23)
Supplement: Figure S4 — IRE1α inhibitors reduce viral protein and are not cytotoxic. [file mbio.00540-23-s0004.pdf]

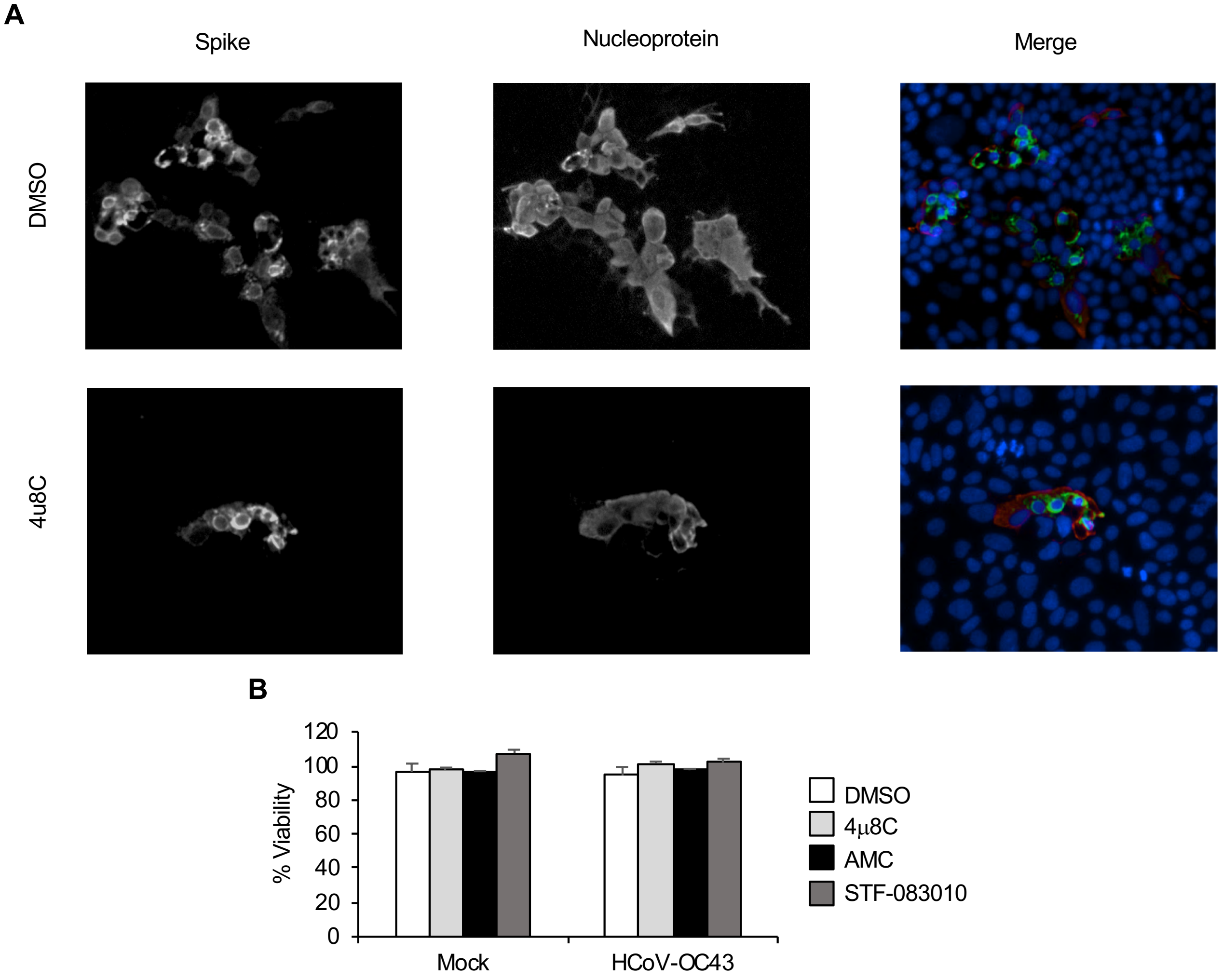

Supplemental Figure 4. **IRE1α inhibitors reduce viral protein and are not associated with cytotoxicity.** **(A-B)** HCT-8 cells were treated with IRE1α nuclease inhibitors or DMSO solvent control prior to infection with HCoV-OC43 at an MOI of 0.01. **(A)** Cells were fixed 48 hours post-infection. HCoV-OC43 viral proteins Spike (green) and Nucleoprotein (red) were visualized by immunostaining. Nuclei were counterstained by DAPI (blue). **(B)** Viability was measured 48 hours post-infection. Data are means ± SD of four replicates **(B)** and are representative of two **(A)** or three **(B)** independent experiments, respectively.
