# Supplementary material for: The unfolded protein response components IRE1α and XBP1 promote human coronavirus infection
Source: mBio. 2023 Jun 12;14(4):e00540-23. doi: 10.1128/mbio.00540-23 (PMC10470493; doi:10.1128/mbio.00540-23)
Supplement: Figure S5 — IRE1α inhibitors decrease SARS-CoV-2. [file mbio.00540-23-s0005.pdf]

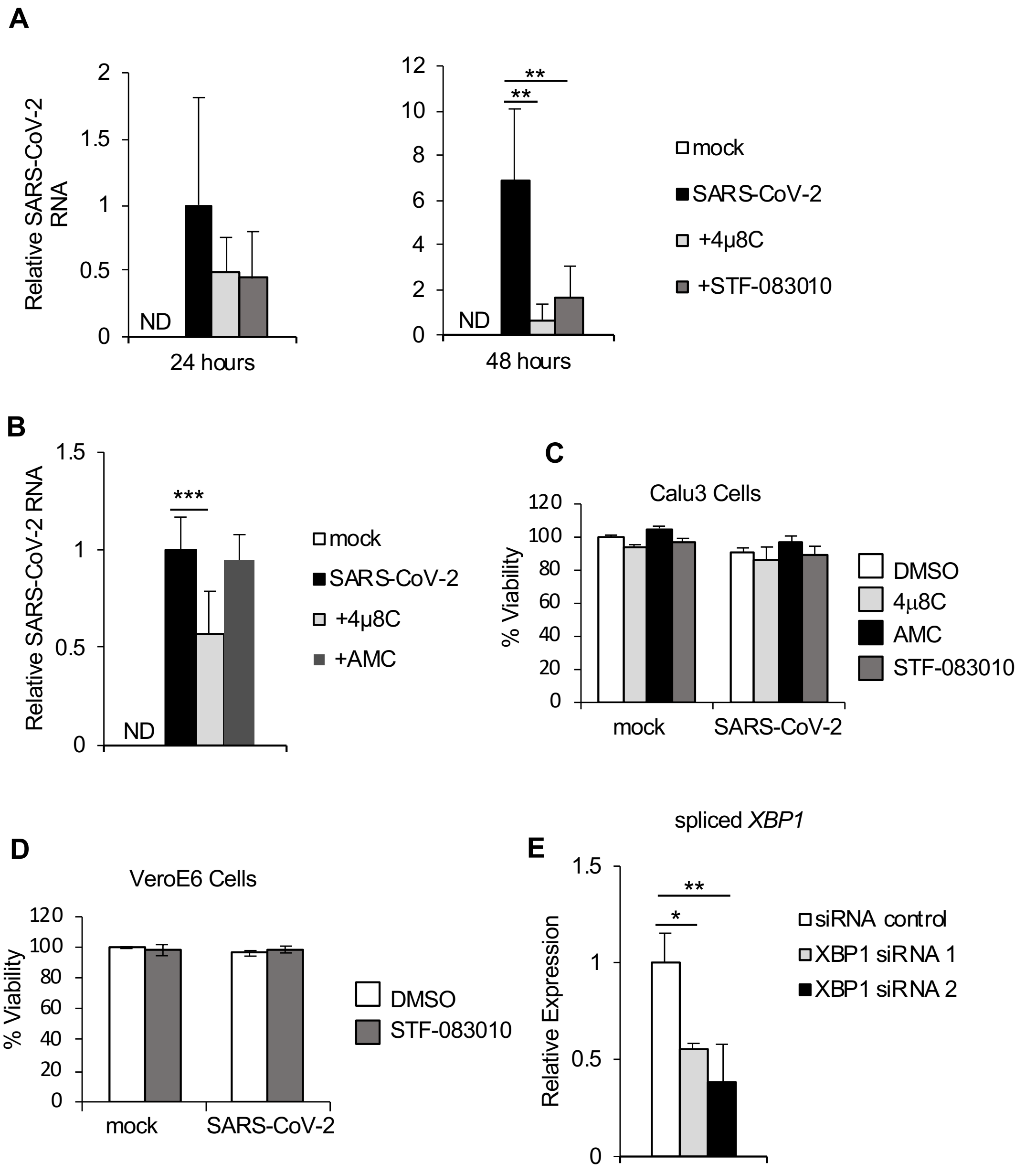

Supplemental 5. **IRE1α inhibitors are not cytotoxic and decrease SARS-CoV-2 viral RNA. (A)** Calu3 cells were treated with IRE1α nuclease inhibitors 4μ8C, STF-0803010 or DMSO solvent control prior to infection with SARS-CoV-2 at an MOI of 0.01. RNA was harvested at 24 and 48 hours post infection and relative abundance of SARS-CoV-2 RNA was determined by RT-PCR. **(B)** Calu3 cells were treated with IRE1α nuclease inhibitor 4μ8C, structurally similar negative control AMC, or DMSO solvent control prior to infection with SARS-CoV-2 at an MOI of 1. RNA was harvested 24 hours post-infection and relative abundance of SARS-CoV-2 RNA was determined by quantitative RT-PCR. **(C)** Calu3 and **(D)** VeroE6 cells were treated with IRE1α nuclease inhibitor 4μ8C, structurally similar negative control AMC, IRE1α nuclease inhibitor STF-083010 or DMSO solvent control prior to infection with SARS-CoV-2. Viability was measured 48 hours post-infection. **(E)** Calu3 cells were transfected with siRNA targeting XBP1 or nontargeting control siRNA prior to SARS-CoV-2 infection and measurement of viral RNA by quantitative RT-PCR. Relative abundant *XBP1s* RNA was quantified by RT-PCR. $^*P < 0.05$, $^{**}P < 0.01$, $^{***}P < 0.001$ by unpaired *t* test.
