# Supplementary material for: The unfolded protein response components IRE1α and XBP1 promote human coronavirus infection
Source: mBio. 2023 Jun 12;14(4):e00540-23. doi: 10.1128/mbio.00540-23 (PMC10470493; doi:10.1128/mbio.00540-23)
Supplement: Figure S6 — Pretreatment with tert-butyl hydroperoxide. [file mbio.00540-23-s0006.pdf]

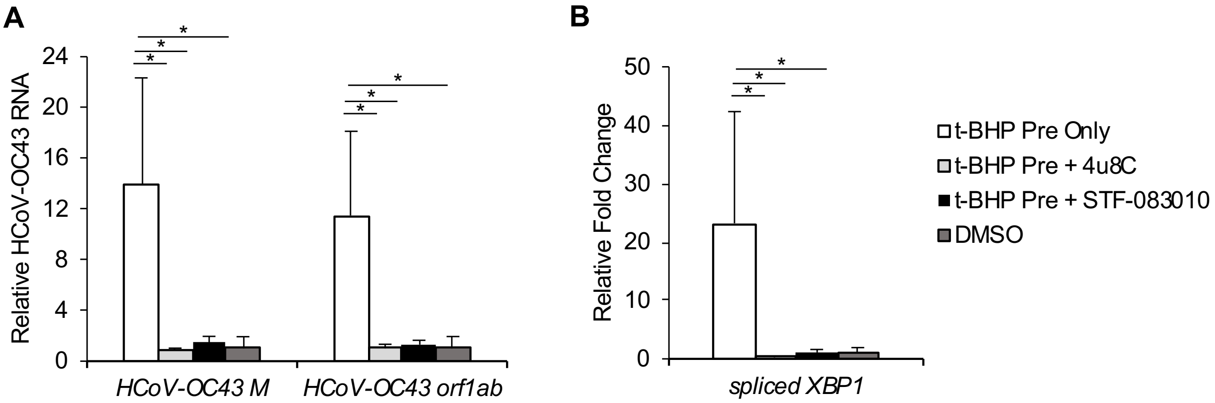

Supplemental Figure 6. **Pretreatment with *tert*-butyl hydroperoxide paired with small IRE1α inhibitor reverses boost in HCoV-OC43 viral RNA production.** HCT-8 cells were treated with *tert*-butyl hydroperoxide (t-BHP) pretreatment alone, t-BHP and small molecule IRE1α inhibitors, or DMSO control 2 hours prior to infection with HCoV-OC43. RNA was harvested 48 hours post-infection and the relative abundance of HCoV-OC43 viral RNA **(A)** and spliced *XBP1* **(B)** were determined by quantitative RT-PCR. Data are means ± SD of four replicates and are representative of two independent experiments.*$P < 0.05$ by unpaired *t* test.
